# Endoscopic Ultrasound-Guided Botox Injection for Refractory Anal Fissure

**DOI:** 10.3390/jcm11206207

**Published:** 2022-10-21

**Authors:** Navkiran Randhawa, Ahamed Khalyfa, Rida Aslam, M. Christopher Roebuck, Mahnoor Inam, Kamran Ayub

**Affiliations:** 1 Department of Internal Medicine, Pacific Northwest University of Health Sciences, Yakima, WA 98901, USA; 2 Department of Internal Medicine, A.T. Still University, Kirksville, MO 63501, USA; 3 Department of Gastroenterology, Franciscan Health Olympia Fields, Olympia Fields, IL 60461, USA; 4 Rx Economics, Hunt Valley, MD 21031, USA; 5 Southwest Gastroenterology, Oak Lawn, IL 60453, USA; 6 Silver Cross Hospital, New Lenox, IL 60451, USA

**Keywords:** EUS, botox, anal fissure, endoscopy

## Abstract

Background: Anal fissures cause severe pain and can be difficult to treat. Medical therapy is initially used, followed by sigmoidoscopy-guided botox injections if the medical therapy is not successful. With this technique, however, it is not clear whether botox is injected into the muscle layer or submucosa. Aim: To evaluate the efficacy of EUS-guided botox injection directly into the internal sphincter. Methods: Consecutive patients with chronic anal fissure refractory to conventional endoscopic botulinum toxin type A injection were enrolled in the study. EUS was performed using a linear array echoendoscope, and a 25 G needle was used to inject botox. All patients were followed up at one- and two-month intervals. Results: Eight patients with chronic anal fissures were included in the study. Six patients had an excellent response to botox at the two-month interval using a visual analog pain scale, while one patient had a moderate response with a pain score reduction of 40%. One patient had no response. No complications were noted. An improvement in visual analog scale (pre-score > post-score) was statistically significant at the *p* < 0.01 level. Conclusion: EUS-guided botox injection into the internal sphincter appears to be a promising technique for patients with refractory anal fissure with pain.

## 1. Introduction

Anal fissures are a well-known disease worldwide. In the United States, approximately 235,000 new cases of anal fissures are diagnosed each year, while in Italy it is the second most common cause of proctologist visits [1,2]. Anal fissure symptoms cause patients significant distress and reduce their quality of life substantially [3]. Although the etiology of anal fissures is controversial, hypertonia of the internal anal sphincter (IAS) has been recognized as a key player in the pathogenesis of the disease. Persistent IAS ischemia and ulceration can lead to severe complications including perianal fistulas, anorectal abscess formation, and anal incontinence [4,5]. 

Thus, treatment for anal fissures is aimed at reducing IAS spasms to relieve pain, decrease ischemia and promote the healing of ulcers. Acute anal fissures are commonly managed by conservative medical treatment, while chronic anal fissures are refractory to such treatment. Surgical treatment, such as sphincterotomy, is commonly required for the treatment of chronic anal fissures or abscesses, providing symptomatic relief [6,7]. However, this procedure requires sphincter injury and has been associated with permanent complications ranging from incontinence of gas in up to 45% of patients to stool incontinence in up to 22% [8,9]. 

Due to such surgical complications, reversible relaxation of the IAS through botulinum toxin type A (BTX) injection has become a common treatment [10]. The injection of type A botulinum neurotoxin produces a constant reduction in maximum resting pressure of IAS and acts like a chemical sphincterotomy. The effect lasts for a few months, giving time for the fissure to heal. Despite being clinically beneficial and causing minimal side effects, achieving proper placement is difficult due to the small target involved [11,12,13]. Ultrasonography has been utilized for direct visual guidance in prior research papers. In 1997, Hofmann et al. reported the first endoscopic ultrasound (EUS)-guided injection of BTX directly into the lower esophageal sphincter muscle as a treatment of achalasia. This treatment proved to be more effective than endoscopic BTX injection without visualization of the direct tissue layers. Our paper similarly explores the utilization of EUS-guided BTX injection directly into the sphincter muscle to treat anal fissures. Our aim is to evaluate the efficacy and safety of EUS-guided BTX injection directly into the internal sphincter in patients with chronic anal fissure refractory to conventional endoscopic botox injection.

## 2. Materials and Methods

### 2.1. Study Patients

Consecutive symptomatic adults with chronic anal fissure refractory to conventional endoscopic four-quadrant BTX injection were enrolled in the study. Refractory was defined by patient’s who failed prior medication and endoscopic botox treatment without the guidance of an endoscopic ultrasound. The inclusion criteria were as follows: (i) evidence of induration in the anal canal, (ii) persistent symptoms of post-defecation/nocturnal pain or bleeding for over 3 months, (iii) failed previous endoscopic injection. 

The exclusion criteria included: acute anal fissure, anal fissure secondary to underlying pathology, known sensitivity to BTX, or patients who were unable to consent to the procedure. 

The study protocol was approved by the Institutional Review Board. 

### 2.2. Operative Technique 

**Figure 1:**
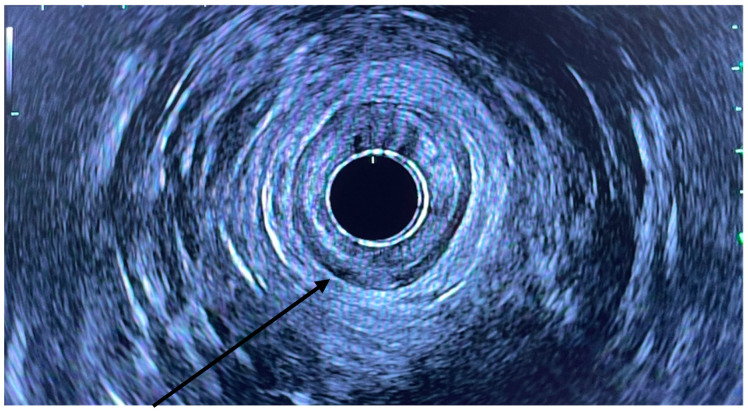
Radial EUS with arrow pointing to IAS.

**Figure 2:**
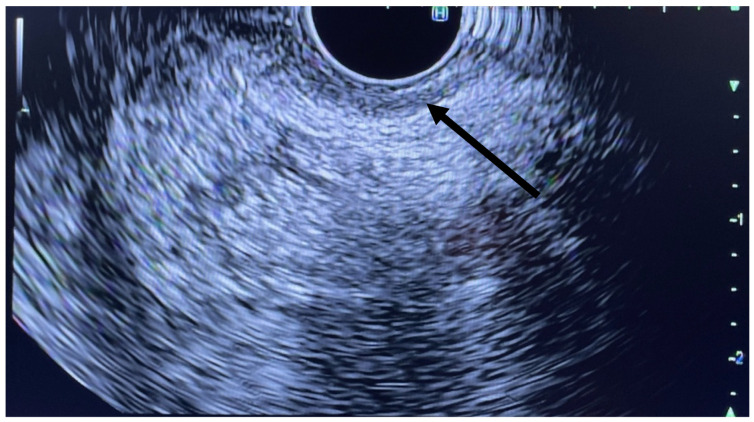
Linear EUS with arrow pointing to IAS.

**Figure 3:**
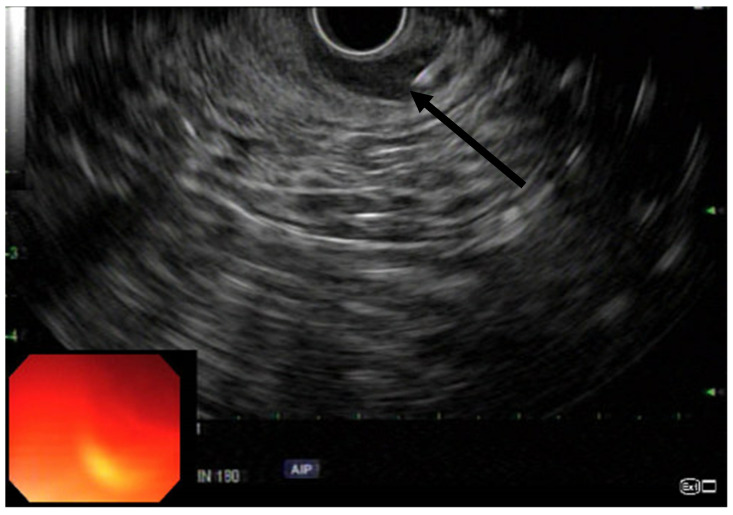
Linear EUS with arrow pointing to the needle injecting the IAS with expansion of the IAS due to the injection.

EUS was performed using a linear array echoendoscope (Figure 1, Figure 2 and Figure 3). Eighty units of type A botulinum neurotoxin was diluted in 2 cc of isotonic saline. An echoendoscope was introduced into the anal canal. The internal sphincter was identified sonographically and a 25-gauge needle was introduced into the internal sphincter. Then, 0.5 cc of saline-containing 20IU BTX was injected into the internal sphincter. The needle was withdrawn, the scope was rotated 90 degrees and the second injection was given. This process was repeated for a total of 4 times giving 0.5 cc per quadrant. Conscious sedation or MAC anesthesia was used for the procedure. 

### 2.3. Clinical Care and Follow-Up 

All patients were followed up at 1- and 2-month intervals by telephone or in person. The patients were asked to determine their pain level by using a 10-point visual analog pain scale. This was compared to the pain score at baseline. 

### 2.4. Statistical Analysis

All statistical analyses were performed using Excel. All results were expressed as the mean +/− standard deviation, and differences between pre- and post-visual analogs were determined via a paired *t*-test. *p*-values of less than 0.05 were considered to be statistically significant. 

## 3. Results

Twelve consecutive patients were assessed for eligibility; of these, four patients did not meet the inclusion criteria. Three patients were unable to consent or refused to consent to participate in the study, and one patient had an acute fissure (not chronic) and superimposed hemorrhoids.

All patients reported severe post-defecation pain. All patients had evidence of a posterior anal fissure from a digital rectal exam and colonoscopy.

We offered EUS-guided BTX injections into the IAS to all patients who presented with anal fissure pain for over 3 months that had been refractory to prior endoscopic BTX treatments. A total of eight patients with prior flexible sigmoidoscopy with BTX injection were included in the study. The outcome was defined as excellent if there was a 50% or greater decrease in the visual analog pain score. Six patients had an excellent response to BTX at the two-month interval using the visual analog pain scale. One patient had a moderate response with a pain score reduction of 40%. One patient, on chronic narcotic treatment, had no response (Table 1). Opioid addiction and opioid-induced hyperalgesia is suspected to explain one patient’s lack of response to chronic opioid therapy. No complications, including incontinence, were reported by the patients after the EUS-guided BTX injection of the internal anal sphincter. The mean pre-treatment visual analog scale score was 9.75, whereas the post-treatment score at week 4 was 5, and 3.8 at week 8. The improvement in this score (pre-score versus post-score) was statistically significant at the *p* < 0.01 level. 

## 4. Discussion

Current therapies for anal fissure include pharmacotherapy, flexible sigmoidoscopy-guided BTX injection and surgical myotomy. In a prior study, Brisinda et al. compared a conservative treatment of 0.2% glyceryl trinitrate ointment to botulinum toxin in patients. This study revealed a 96% healing rate in the botulinum toxin group, compared with 60% in the glyceryl trinitrate group [12]. Thus, medical therapy alone with topical agents including nitrates can be relatively ineffective for chronic anal fissures [14]. On the other hand, surgical myotomy has the potential to offer long-term benefits, but it carries the risk of complications [15]. Alternative treatments are needed for patients with comorbidities or advanced age. The injection of BTX into the IAS has been shown to treat refractory anal fissure with good outcomes [10]. Maria et al. in their study found the local injection of botulinum toxin into the IAS to be a promising approach to the treatment of anal fissures [10]. However, this procedure when completed blindly can be technically challenging, as it relies on the endoscopist’s tactile sense for the proper placement of the needle into the IAS. Cagri et al. further described the efficacy and safety of endoanal ultrasound (EAUS)-guided botulinum toxin in the treatment of chronic anal fissure [16]. The study revealed that the efficacy rate was higher in the EAUS group, but these results were not statistically significant.

In this pilot case series, we examined the utility of EUS-guided BTX injection into the IAS under direct visualization in patients with anal fissures refractory to medications and endoscopic therapies. Consecutive patients in our study who underwent EUS-guided BTX injection had excellent responses with a reduction in pain score with no complications. Furthermore, a statistically significant improvement in the visual analog scale was seen.

While, to our knowledge, our study is the first case series to report on EUS-guided botulinum injection for refractory anal fissures, we recognize several limitations. First, patients’ inclusion and response assessment was based on some subjective criteria which are difficult to quantify and, therefore, might potentially introduce bias into our results. Second, the sample size was small, and thus our results may not be generalizable or applicable to larger populations. In addition, because our sample size was small, we cannot exclude the presence of selection bias in our study. Finally, the follow-up time was short and, therefore, no firm conclusions can be drawn from our results on long-term outcomes of this procedure and related complications. Furthermore, large multicenter studies will be needed to address these limitations.

## 5. Conclusions

In summary, EUS-guided BTX injection is a promising technique for patients with anal fissure refractory to medical therapy and appears to be superior to endoscopic BTX injection without ultrasound guidance. More studies need to be conducted to confirm the efficacy of this approach. 

## Figures and Tables

**Table 1:** Study patients.

Patients	Age (y)/Sex	Initial Visual Analog Pain Score	Prior Failed Therapy	Visual Analog Pain Scale Improvement 1 Month after Procedure	Visual Analog Pain Scale Improvement 2 Months after Procedure	Final Outcome
1	22/M	10	Medication + Endoscopic botulinum toxin (BTX)	5	3	Excellent Response
2	33/M	10	Medication + Endoscopic BTX	4	3	Excellent Response
3	48/F	10	Medication + Endoscopic BTX	3	3	Excellent Response
4	61/F	10	Medication + Endoscopic BTX	4	2	Excellent Response
5	42/F	9	Medication + Endoscopic BTX	4	2	Excellent Response
6	60/M	9	Medication + Endoscopic BTX	4	2	Excellent Response
7	40/F	10	Medication + Endoscopic BTX	6	6	Moderate Response
8	48/M **	10	Medication + Endoscopic BTX	10	10	No response

** Patient was on narcotic pain medications from an outside clinic. Excellent response = 50% or greater decrease in the visual analog pain score. Moderate response = pain score reduction of 40%. No response = 0% change in pain score.

## Data Availability

Not applicable.
